# Shortcomings and missed potentials in the management of migraine patients - experiences from a specialized tertiary care center

**DOI:** 10.1186/s10194-019-1034-8

**Published:** 2019-08-01

**Authors:** Christian Ziegeler, Greta Brauns, Tim P. Jürgens, Arne May

**Affiliations:** 1 Department of Systems Neuroscience, University Medical Center Hamburg-Eppendorf, Martinistr. 52, 20246 Hamburg, Germany; 2 Department of Neurology, University Medical Center Rostock, Rostock, Germany

**Keywords:** Migraine, Care, Management, Guideline

## Abstract

**Background:**

Migraine is a common and severely disabling neurological disorder affecting millions of patients in Europe. Despite the availability of evidence-based national and international guidelines, the management of migraine patients often remains poor, which is often attributed to a low availability of headache specialists. The aim of this study was to investigate the adherence to national guidelines and to assess the possible potential of optimized therapy regimens in migraine patients.

**Methods:**

We collected data of migraine patients presenting to our out-patient clinic via standardized questionnaires regarding headache, diagnostics and experience with previous treatments. We also assessed the efficacy of treatment started by our center.

**Results:**

1,935 migraine patients were included between 2010 and 2018. In the 12 months before consulting our headache clinic 89.5% of the patients had consulted a general practitioner and 74.9% had consulted a neurologist because of their migraine. Nevertheless, 50% of the patients underwent unnecessary diagnostics and 34.2% had not been treated according to evidence-based treatment guidelines. Out of 1,031 patients who had not been prescribed a preventative treatment 627 (60.8%) had in average 3 or more migraine attacks per month and thus qualified for a preventative treatment. These patients missed in the 3 months prior to consultation on average 5 work or school days. Initiating a preventative treatment was effective in 71.2% of the patients, that provided follow-up data.

**Conclusions:**

Our data suggest, that many migraine patients to this day do not receive state-of-the-art therapy. Adherence to national and international European guidelines could improve the outcome in migraine patients. Future research should try to answer why guidelines are not followed.

## Introduction

Migraine is a common and severely disabling neurological disorder affecting millions of patients [1] and due to its high prevalence it is considered to be one of the most disabling diseases worldwide [2]. The prevalence in Europe is estimated to be around 15% [3]. Even though costs-per-subject are not as high as in other neurological and central nervous system diseases, the combined direct and indirect annual costs of migraine in the European Union accounted for €111 billion in a 2011 study [4].

Despite the availability of evidence-based national [5] and international [6] guidelines, the management and care provided to migraine patients is far from ideal [7]. Migraine patients still experience significant time delays in the diagnosis of their headache disorder [8] and the majority of migraine patients seem to not receive adequate acute [7] and preventative treatment [9]. In this context it has been suggested that the diagnosis of migraine and its implications are still not fully recognized by the patient and especially by the medical community [10]. However, patients referred to a 3rd referee center (tertiary care center) such as University headache outpatient clinic are usually already diagnosed and treated by general practitioners and neurologists and should therefore represent a group of patients who are particularly hard to treat. Since the headache syndromes of these patients are per definition recognized by the patient and by the medical community, the question arises whether these patients were treated correctly or not, i.e. whether these patients suffer from particularly severe headache disorders and therefore are medically intractable or whether they have not been treated properly despite recognition of the disease. The aim of the current study is therefore to investigate the adherence to national migraine guidelines for diagnosis and medical treatment in a sample of nearly 2000 patients consulting a third referee center for headache.

## Methods

This study was approved by the local ethics committee of the chamber of physicians of Hamburg, Germany (PV 3185) and patients gave written informed consent according to the Declaration of Helsinki.

The headache and facial pain outpatient clinic is a specialized tertiary care center within the University Medical Center Hamburg-Eppendorf with approximately 1000 to 1200 annual registered patient contacts. These contacts reflect all combined consultations by migraine patients, patients with other primary or secondary headaches as well as patients suffering from primary and secondary facial pain syndromes. It must be noted, that multiple personal contacts (even for prescriptions) by the same patient are respectively counted as additional contacts due to the nature of German reimbursement policy. Due to German data protection law, data from patients not willing to participate in this study was not saved and we are therefore not able to provide a complete overview over all patients who consulted us.

Our outpatient clinic is specifically designed to be consulted by patients in whom the general practitioner or other experts such as neurologists ask for a second opinion or a therapy optimization, however also self-referrals by patients are possible and not separately counted. We usually recommend adjusting therapies in accordance to the national German guideline for the acute and preventative treatment of migraine, with a focus on implementing pharmacological and non-pharmacological treatment regimens [5].

Before the first consultation, patients are asked to voluntarily fill out a custom-built electronic questionnaire (AC-STB, http://www.ac-stb.de/de/) to provide personal sociodemographic data, description of disease specific symptomatology, health care utilization, as well as other standardized questionnaires such as the MIDAS [11]. Medical records of the consultation and a questionnaire filled out by the clinicians are linked to the data. Data are stored pseudonymized and for the evaluation of this study they were anonymized.

Clinicians focus on additional disease characteristics and especially previous treatment strategies, including description and evaluation of the pharmacological treatment. The combination of these questionnaires allows for comprehensive and multi-dimensional data acquisition.

Only correctly and duly completed data sets by all patients willing to participate are included into the database. Exclusion criteria were patients refusal to participate in the questionnaire and patients not fitting ICHD-3 criteria [12] for migraine.

Consecutive consultations are also documented with a questionnaire designed to document adherence to medical advice and treatment as well as efficacy and tolerability of such treatments. Headache days are usually evaluated calendar-based, but some patients refrain from using a calendar. In these cases, we rely on the patients’ oral information.

Since migraine comprises the largest group of patients, providing meaningful data, we focused on migraine according to ICHD-3 criteria [12] in the following. Of note, for all our parameters that we report we focused on the first contact with the patients to get a clear picture what type of patients consult a university outpatient specialist clinic. Only regarding preventative efficacy of medications we prescribed, we looked into follow-up visits. Original data are from 1935 distinct patients.

## Results

Between the years 2010 and 2018 1,935 migraine patients provided complete data sets. This corresponds to a rate of approximately 75% of all distinct migraine patients (including possible and atypical cases, hence the uncertainty) seen during this time interval. Of these 1,578 (81.6%) were female, and 357 patients (18.4%) were male. The mean age at the first consultation was 37.3 ± 13.3 (SD) years (unknown date of birth in two female patients).

### Headache days

Migraine patients suffered 12.1 ± 9.6 headache days per month (*n* = 1,879 patients, inconclusive data for 56 patients). We further divided our patients into subgroups (Table 1) of:

(i) 1–3 headache days/month
(ii) 4–14 headache days/month
(iii) chronic with 15 or more headache days/month

**Table 1:** All migraine patients grouped by sex, amount of migraine days, number of attacks and previous preventative treatments in their lifetime, overall (*n* = 1,935). n/a: not applicable

Group	Overall (*n* = 1935)	Female (*n* = 1578)	Male (*n* = 357)
(i) 1-3 days	367 (19.0%)	286 (18.1%)	81 (22.7%)
(ii) 4-14 days	949 (49.0%)	790 (50.1%)	159 (44.5%)
(iii) chronic (15 days or more)	563 (29.1%)	451 (28.6%)	112 (31.4%)
n/a	56 (2.9%)	51 (3.2%)	5 (1.4%)
	At least one preventative treatment	No prior preventative treatment	
overall	904/1935 (46.7%)	1031/1935 (53.3%)	
(iv) ≥3 attacks	672 (74.3%)	627 (60.8%)	
(v) <3 attacks	209 (23.1%)	371 (36.0%)	
n/a	23 (2.5%)	33 (3.2%)	

as well as:

(iv) ≥3 attacks/month and
(v) < 3 attacks/month

In chronic migraine patients, migraine and headache days were not separated for this analysis.

### Medication overuse

At the time of the first consultation 9.2% (179/1,935) of all patients fulfilled the ICHD-3 criteria of medication-overuse headache. Of these, 77.1% (138/179; not specified: 2) had never undertaken a withdrawal.

In total, a withdrawal from headache medication had been undertaken by 11.8% of all patients (229/1,925 patients, not specified: 10) prior to the first consultation. Of those 38.0% were hospitalized and 59.0% underwent withdrawal as outpatients (222; not specified: 7). These lifetime data combine self-initiated withdrawals, as well as withdrawals suggested by doctors.

### Health care utilization

**Fig. 1:**
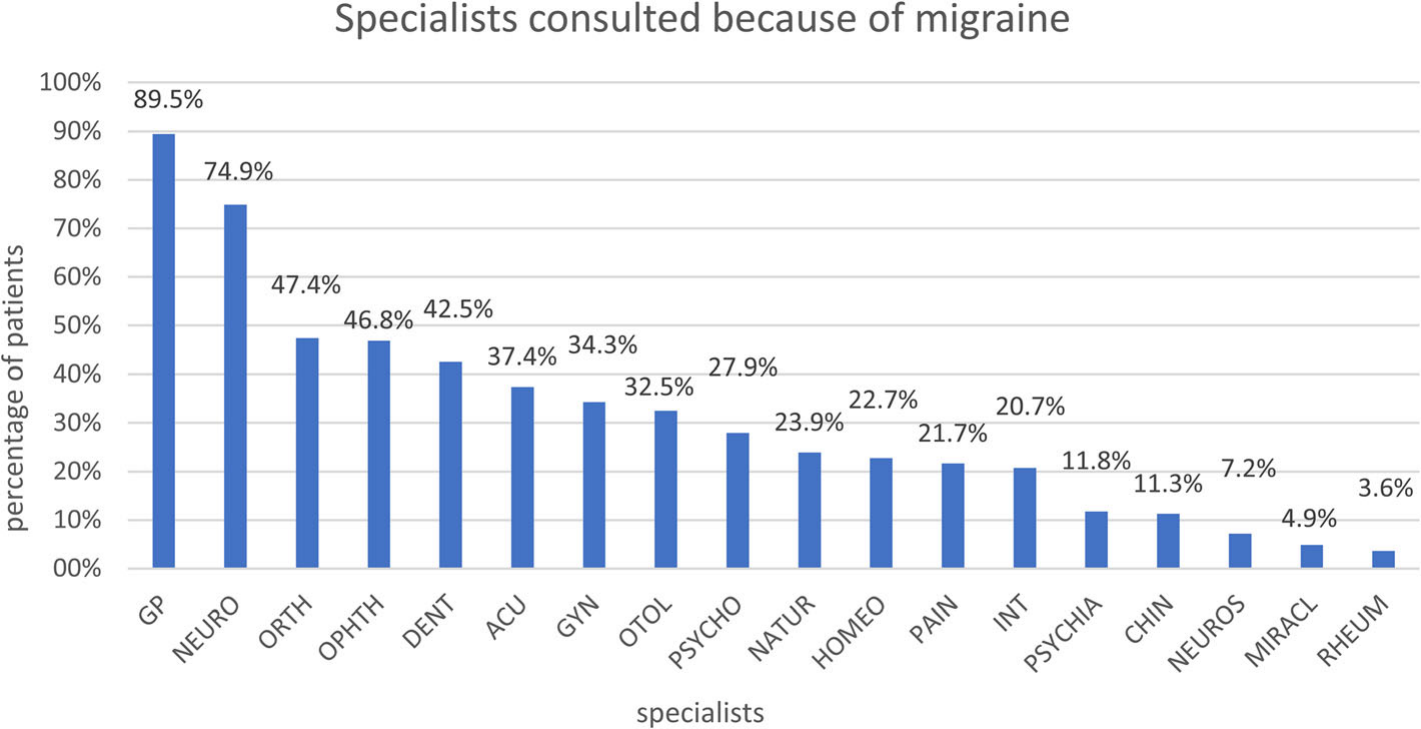
Specialists which were consulted by patients because of their migraine (GP: general practitioner; NEURO: neurologist; ORTH: orthopedist, OPHTH: ophthalmologist, DENT: dentist, ACU: acupuncture specialist, GYN: gynecologist, OTOL: otolaryngologist, PSYCHO: psychologist, NATUR: natural medicine, HOMEO: homeopath, PAIN: pain specialist, INT: internist, PSYCHIA: psychiatrist, CHIN: traditional Chinese medicine specialist, NEUROS: neurosurgeon, MIRACL: faith healer, RHEUM: rheumatologist (multiple answers possible). (n = 1,935)

Patients had an average of 7.2 ± 10.0 outpatient consultations because of their migraine in the last 12 months before the initial consultation (*n* = 1,927, *n* = 8 missing data). Most of the patients had consulted a general practitioner (89.5%) followed by neurologists (74.9%) and orthopedists (47.4%) (see Fig. 1).

Throughout their lifetime, 54.2% (1,048/1,935) of the patients underwent a computer tomography (CCT) because of their headache and 51.4% (*n* = 995/1,935) received an MRI of the head, none of which changed the diagnosis.

Nearly one third of all patients reported to have at least once presented to an emergency department because of migraine (31.3%, *n* = 605/1,935) and these patients had presented themselves in average 4.1 ± 7.4 times in total. In-patient stays were also common: 22.5% (*n* = 435/1,935) of patients had previously been hospitalized due to migraine headaches (2.9 ± 5.8 in-patient treatments in these patients in average).

### Previous preventative treatment

An important, effective and often overlooked aspect of the preventative treatment of migraine are non-pharmacological [13] strategies, including endurance and relaxation training as well as biofeedback, lifestyle modifications and possibly specialized cognitive behavior therapy as listed by the German migraine treatment guideline [5]. For this study however, we focus on pharmacological interventions.

The first line pharmacological preventative treatments proposed by the German guideline for the treatment of migraine are beta blockers (metoprolol, propranolol), calcium channel blockers (flunarizine), anticonvulsants (topiramate, valproate) and tricyclic antidepressants (amitriptyline). A preventative is considered necessary when the patient suffers more than 3 migraine attacks per months which affect the quality of life, when attacks consistently last longer than 72 h or do not respond to acute treatment [5].

More than one third of the patients (34.2%, *n* = 661/1,935) have not been treated according to the German guideline for the treatment of migraine. Only 0.6% of our patients (11/1,935) had tried all recommended guideline treatment strategies and were therefore considered medically intractable.

More than half of the patients (53.3%, *n* = 1,031/1,935) had either never been prescribed a preventative treatment or, if described, have not taken it (Table 1). 60.8% (627/1,031) of these patients had in average 3 or more migraine attacks per month (Table 1) and thus qualified for a preventative treatment. Only 36.0% (371/1,031) of the patients with no prior preventative treatment had in average less than 3 migraine attacks per month (inconclusive data for 3.2% [33/1,031]) (Table 1). Patients with no prior preventative treatment missed in the 3 months prior to their first consultation on average 5.1 ± 10.9 (SD) work or school days (MIDAS, Item 1), could not do household work on 8.2 ± 11.1 days (MIDAS, Item 3) and missed on an average of 7.3 ± 11.0 days family or leisure activities (MIDAS, Item 5) because of their migraine headaches.

Patients with no prior preventative treatment and more than 3 migraine attacks per month had consulted a genral practitioner in 87.9% (551/627) of the casesand a neurologist in 67.5% (423/627) of the cases.

Of 904 patients who used preventative medications prior to their consultation, 45.8% had one, 21.2% had two and 10.3% reported three unsuccessful treatment attempts. Successful treatments were reported by 22.5% (203/904) of the patients with one, 3.9% (35/904) with two and 0.8% with three preventatives. Success was defined that a given preventative treatment reduced the headache frequency by at least by 50% and was well tolerated. The clinicians documented unsuccessful treatments when the medication was ineffective or not well tolerated.

### Potential of preventative medication when used following guideline therapy

One third of the patients consulted us for at least one more time (33.4%, 647/1,935). 17.9% (346/1,935) were seen twice, and 10.7% (207/1,935) of patients consulted us three or more times after the first consultation. For these consultations the evaluation of the overall therapy success as well as the success of the acute and preventative pharmacological treatments were assessed. In most cases the second consultation took place between 3 and 6 months after the first consultation.

**Fig. 2:**
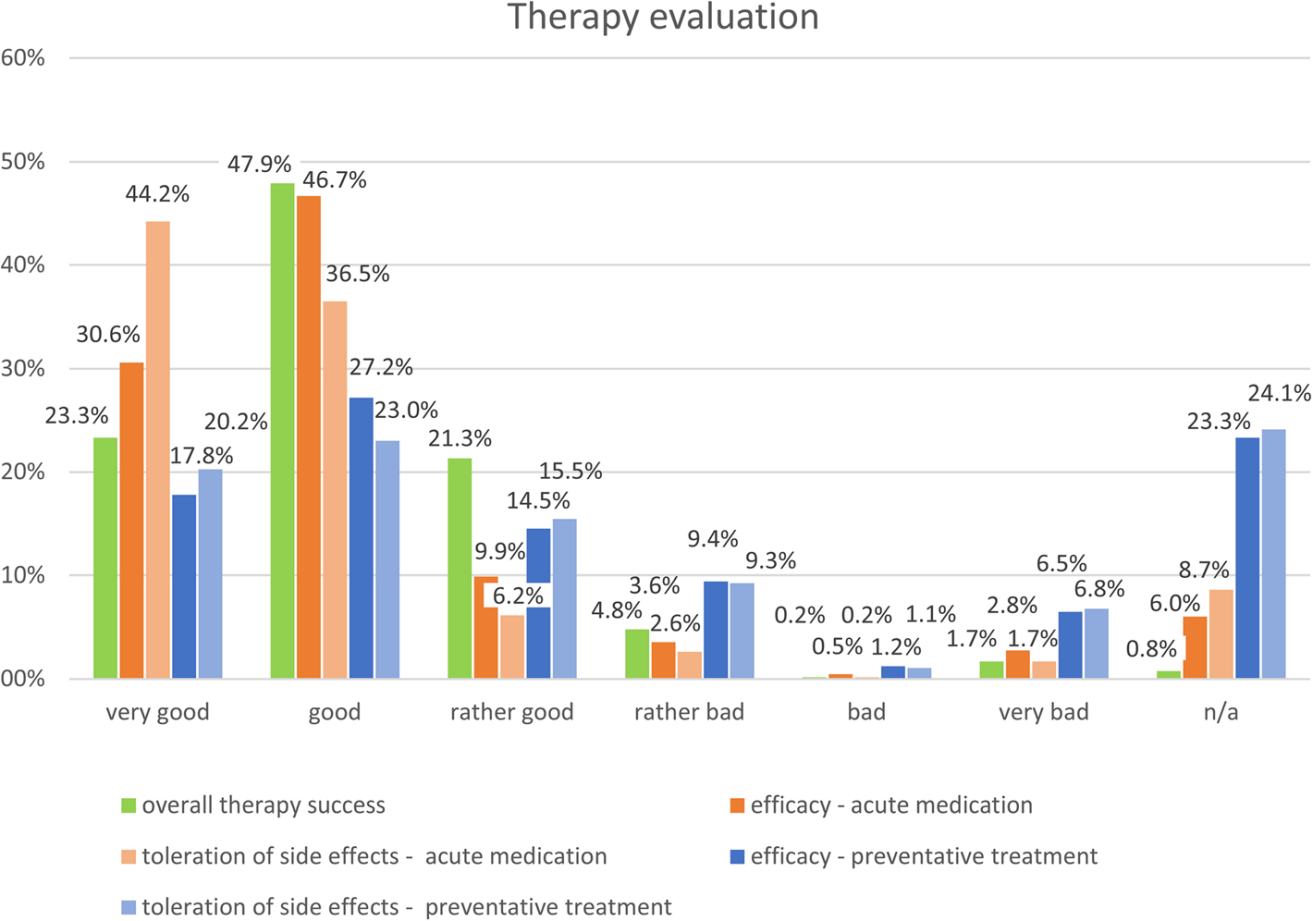
Therapy evaluation of migraine patients presenting for a second time to the out-patient clinic. Clinicians’ evaluation of overall success as well as evaluation of acute and preventative treatment regarding efficacy and tolerability. (*n* = 647). n/a: not applicable

Figure 2 shows evaluation of 647 patients that consulted the out-patient clinic for a second time. Usually these patients were highly affected by their migraine headaches and previous preventative treatment had been unsuccessful or never tried. Efficacy and tolerability were retrospectively rated in an ordinal rating scale by the clinicians with the possible answers ‘very good’, ‘good’, ‘rather good’, ‘rather bad’, ‘bad’, ‘very bad’. The overall success of the therapy initiated in our outpatient clinic was evaluated as very good or good in 71.2% of the cases. Acute treatment showed a very good or good efficacy in 77.3% of the cases and an even better tolerability. The preventative treatment was for the most part effective (59.5% either ‘very good’, ‘good’, or ‘rather good’) and well tolerated (58.7% either ‘very good’, ‘good’, or ‘rather good’).

**Fig. 3:**
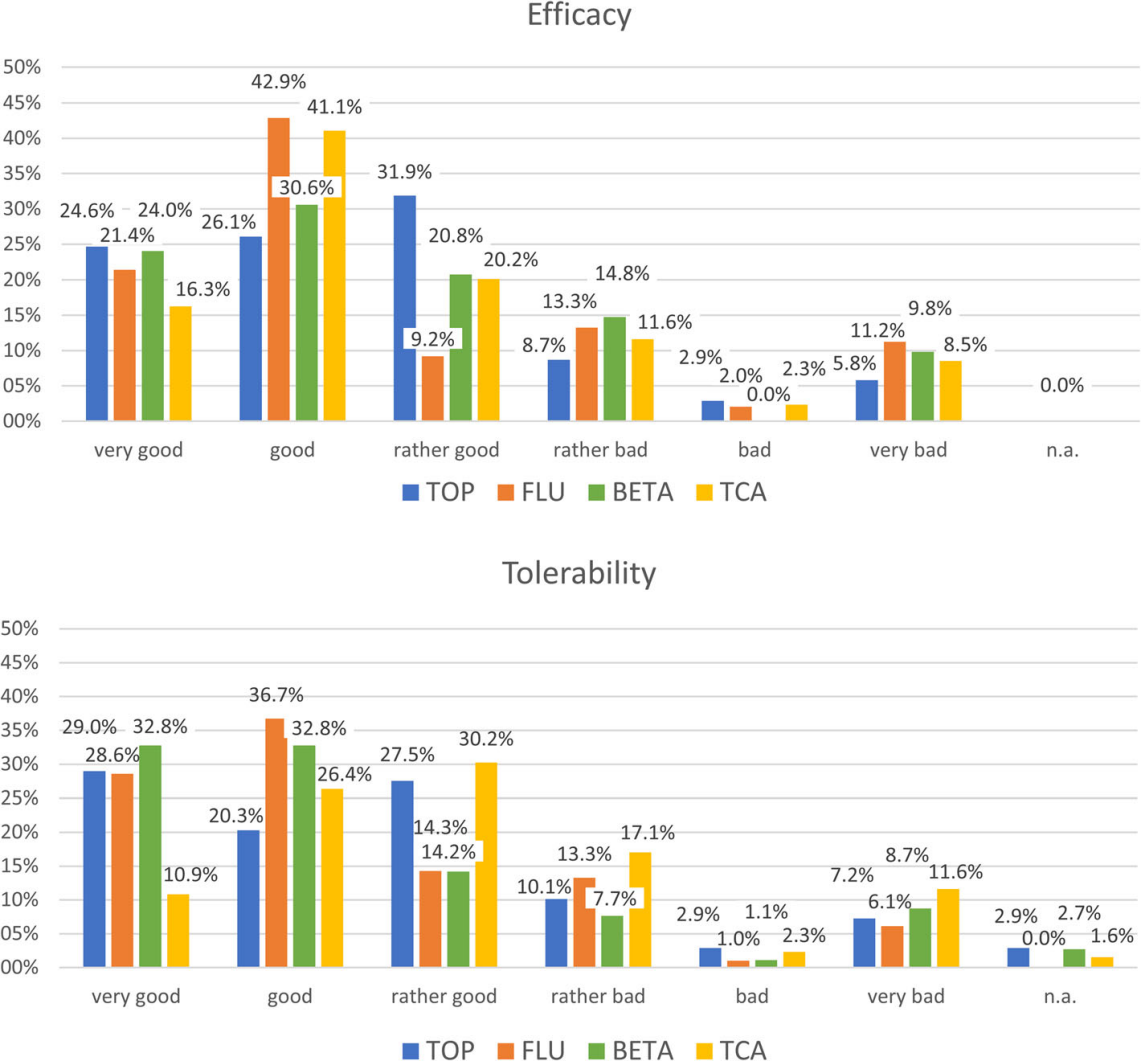
Efficacy and tolerability of the four first-line preventative migraine medications as evaluated by the clinicians during the second consultation (3–6 months after first consultation). TOP: topiramate, FLU: flunarizine, BETA: beta blockers, TCA: tricyclic antidepressants. (*n* = 647). n/a: not applicable

The efficacy and tolerability rates for each of the four most often prescribed first-line preventative drugs topiramate, flunarizine, beta-blockers and amitriptyline are shown in Fig. 3. The results of all substances for efficacy and tolerability are comparable, however there is a trend towards more side effects of amitriptyline compared to the other three.

**Fig. 4:**
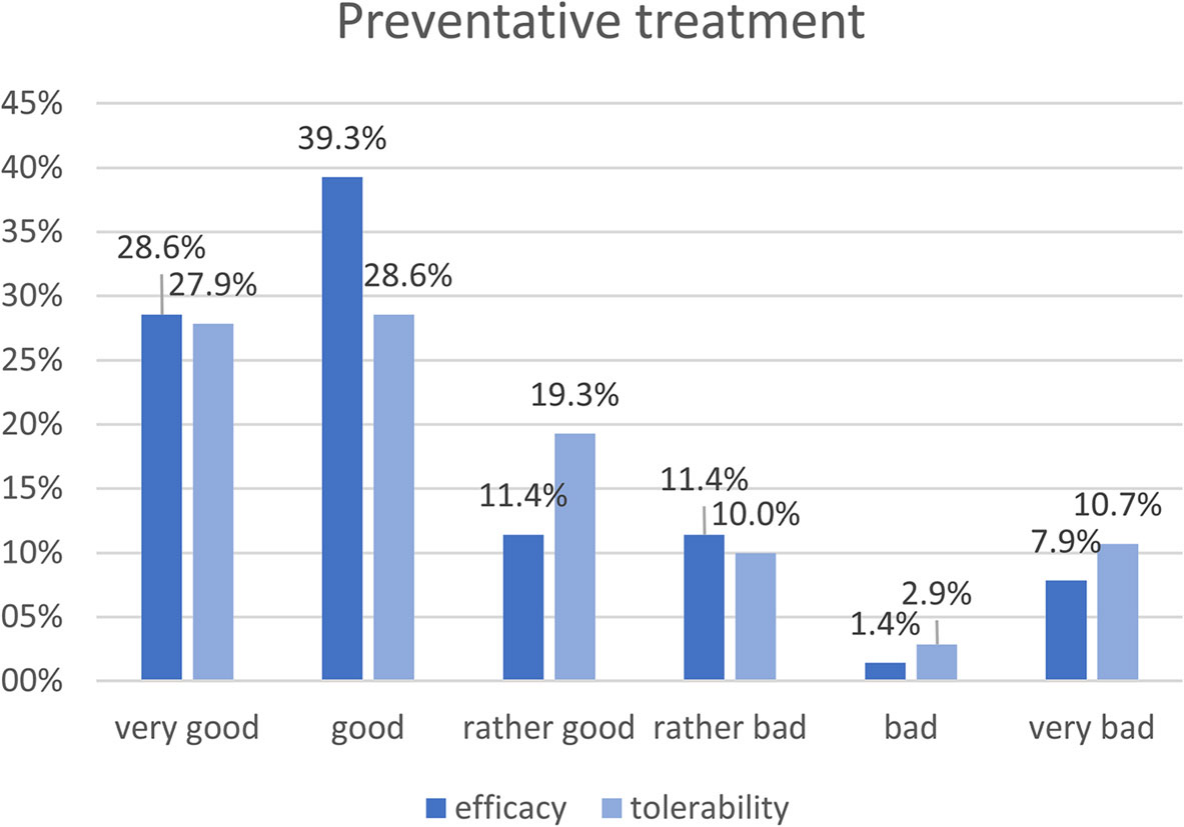
Efficacy and tolerability of preventative treatments in general in patients that had more than 3 attacks per month but had previously not been prescribed a preventative migraine treatment. (*n* = 140)

Overall, we have data for 190 follow-up patients who previously had not been prescribed a preventative treatment even though they had more than 3 attacks per month. Figure 4 shows the efficacy and tolerability rates for 140 out of these patients, in whom a preventative treatment had been prescribed and tried (the other 50 patients did not want to try a preventative for different reasons). The efficacy of preventative treatments in these patients was ‘very good’, good’ or ‘rather good’ in 79.3% of the cases, the tolerability was similar with 75.8% of the cases being ‘very good’, ‘good’ or ‘rather good’ (Fig. 4).

## Discussion

Our data suggest that to this day a significant percentage of migraine patients might not be introduced to state-of-the-art pharmacological therapy as lined out in national guidelines. Given that most patients improve clinically, when we treated pharmacologically according to guidelines, the low adherence to evidence-based treatment strategies has considerable, and more importantly, avoidable personal as well as socio-economic consequences. It is possible that the majority of migraine patients is treated well by their GP and other specialists and that the small percentage of patients presenting to a specialized headache center is not representative for all migraine patients. This possibility is worth considering, however not only our personal experience and this data, but also previous studies strongly indicate, that there are unmet needs for migraine patients [7].

The German Migraine and Headache Society (DMKG) has been founded in 1979 with the aim to foster research and treatment in headache. Since then they publish regular and updated evidence based treatment guidelines [5]. Additionally, in partnership with the German Neurological Society (DGN) there are updates and refreshers on the treatment of migraine patients implemented into national and international conferences and meetings as well as innumerable regional smaller seminars. Moreover, all treatment guidelines are freely available to all readers, not restricted to doctors, in the internet (www.dmkg.de). Despite this abundant supply of knowledge, migraine patients see several doctors and specialists because of their headaches, undergo diagnostic procedures including CT and MR-imaging which are mostly not indicated [14] and are not treated properly. Of note, we only report patients who consulted the headache clinic of the Hamburg University Medical Center and one could argue that patients who have been treated properly escape us, thus overestimating the negative results. This study is not powered or designed to reflect the amount of patients who are treated properly by primary or secondary care specialists, however as mentioned the experience of many headache specialists and literature suggests that many migraine patients are treated poorly [7].

It is striking that we found the prior treatment as not in line with the official guidelines in over a third of all patients. This problem of sub-optimal adherence to national guidelines in headache has been discussed before [15]. Also, half of the patients, presenting to a specialized tertiary care headache clinic, had never been prescribed a preventative treatment. The data shows that over 60% of these patients should have received a preventative treatment (Table 1) and additionally indicates that many of these patients potentially profit from it (Fig. 4).

A limitation of our study approach is that we cannot provide follow up data for all patients and therefore we acknowledge that especially the effect of the preventative treatments proposed by us has to be viewed critically. This study reflects the clinical reality of treating migraine patients in a tertiary care setting. Patients are offered to present themselves again in case they do not profit from the recommended therapy. Many patients especially with positive outcomes however do not consult us again but are subsequently treated by their primary care physicians again. There are patients who only consulted us once, which were probably inadequately treated or suffer side-effects and are therefore lost for follow-up. However, the rate of patients who cancel a consultation is with 10% of patients remarkably stable since years (data collected on a monthly basis), despite a more than 90% rate of all patients who would recommend and consult us again (data collected 4 weeks after consultation by phone). Still, it is possible that we overestimate the success rate of the preventative treatment initiated by us to a certain extent and we acknowledge this fact. The rates of the improved outcome in patients that had previously not been prescribed a preventative treatment should therefore be seen as an indication that adherence to guideline therapy possibly improves the outcome, as expected.

At the same time, although unlikely, we cannot rule out that we overestimate the adherence to guidelines since some patients may have, for example, a beta blocker intended for the treatment of hypertension or amitriptyline intended for the treatment of depression and we documented it as under the migraine indication. We also note that clinical experience and also recent studies show that the adherence to oral preventative medication, especially in chronic migraine is low [16]. This is most likely owed to insufficient success combined with problematic side effects.

In conclusion, this study shows the importance to better implement treatment guidelines into day-to-day clinical practice. Additionally, headache education should play a larger role in medical school for young doctors. It is the authors’ experience in teaching headache to students, that despite its relevance, headache only plays a minor role in the curricula. This also holds true internationally [17], however useful curricula have been proposed [18]. We know that inadequate acute treatment leads to migraine chronification [19] and this certainly also holds true for insufficient preventative treatment [20]. While it is understandable that highly consulted specialists underlie time constraints, it is not easily explainable why many patients who qualify for preventative treatment have not been prescribed one. Given the statutory health insurance fund costs for consultations, diagnostics and treatments it is rather alarming that a considerable amount of these costs (specialist consultations, emergency admittance, MR and CCT imaging, OTC medications, non-pharmacological treatment attempts etc.) are either not necessary or may even be contra-productive. We lack information about the decision processes made by primary-care physicians with regard to the treatment of migraine. Many of these patients, especially with episodic migraine, profit greatly from first line preventative drugs. Further research should try to highlight, why the adherence to guidelines in daily clinical practice is low. Possible factors could be that clinicians are unaware of the applicable guidelines, but also that medications are not well tolerated by patients and therefore discontinued.

## Data Availability

All patients who participated in this study gave written informed consent. However, this consent did not include a provision stating that individual raw data can be made freely accessible to the public. Therefore, in accordance with the German data protection act §4 the underlying raw data cannot be made accessible to the public. Researchers meeting the criteria for access to confidential data may access anonymized data upon request, involving the documentation of data access.
